# Evaluation of the antitumor effect of neoantigen peptide vaccines derived from the translatome of lung cancer

**DOI:** 10.1007/s00262-024-03670-0

**Published:** 2024-05-14

**Authors:** Fenbao Lian, Haitao Yang, Rujun Hong, Hang Xu, Tingting Yu, Gang Sun, Guanying Zheng, Baosong Xie

**Affiliations:** 1 Shengli Clinical Medical College, Fujian Medical University, No. 134 East Street, Fuzhou City, 350001 Fujian Province China; 2 Department of Respiratory Medicine and Critical Care Medicine, Fujian Provincial Hospital, No. 134 East Street, Fuzhou, 350001 China; 3 Department of Thoracic Oncology, The Affiliated Tumor Hospital of Xinjiang Medical University, Urumqi, 830011 Xinjiang China; 4 Department of Breast and Thyroid Surgery, The Affiliated Tumor Hospital of Xinjiang Medical University, 789 East Suzhou Street, Xinshi District, Urumqi, 830011 Xinjiang China; 5 Xinjiang Cancer Center/Key Laboratory of Oncology of Xinjiang Uyghur Autonomous Region, Urumqi, 830011 Xinjiang China

**Keywords:** Neoantigen, Translatome, Immunotherapy, Lung cancer, Peptides

## Abstract

Emerging evidence suggests that tumor-specific neoantigens are ideal targets for cancer immunotherapy. However, how to predict tumor neoantigens based on translatome data remains obscure. Through the extraction of ribosome-nascent chain complexes (RNCs) from LLC cells, followed by RNC-mRNA extraction, RNC-mRNA sequencing, and comprehensive bioinformatic analysis, we successfully identified proteins undergoing translatome and exhibiting mutations in the cells. Subsequently, novel antigens identification was analyzed by the interaction between their high affinity and the Major Histocompatibility Complex (MHC). Neoantigens immunogenicity was analyzed by enzyme-linked immunospot assay (ELISpot). Finally, in vivo experiments in mice were conducted to evaluate the antitumor effects of translatome-derived neoantigen peptides on lung cancer. The results showed that ten neoantigen peptides were identified and synthesized by translatome data from LLC cells; 8 out of the 10 neoantigens had strong immunogenicity. The neoantigen peptide vaccine group exhibited significant tumor growth inhibition effect. In conclusion, neoantigen peptide vaccine derived from the translatome of lung cancer exhibited significant tumor growth inhibition effect.

## Introduction

Lung cancer stands as the most malignant neoplasm globally, characterized by its high malignancy, rapid progression, therapeutic challenges, and poor prognosis. Currently, immunotherapy for cancer primarily revolves around immune checkpoint inhibitors, especially PD-1/PD-L1 inhibitors. Clinical studies have demonstrated that PD-1/PD-L1 inhibitors exhibit sustained response rates in the realm of lung cancer treatment and improve substantially the survival rates of advanced cancer patients by disrupting the PD-1/PD-L1 binding and relieving immune suppression. However, primary resistance occurs in 7–27% during first-line treatment and 20–44% during second-line treatment within the subset of lung cancer patients receiving immunotherapy [1]. Furthermore, results from the KEYNOTE-001 study indicate that approximately 25% of patients develop acquired resistance [2]. Although immunotherapy checkpoint inhibitors display a notable survival advantage in clinical research when compared to chemotherapy, the objective response rate (ORR) in unselected patients remains around 20% [3–5]. Consequently, the exploration of novel treatment modalities becomes increasingly imperative.

Neoantigens, with their high specificity confined to tumor tissues and absence in normal tissues, evade central immune tolerance and exhibit heightened safety, rendering them ideal targets for cancer immunotherapy. With the advancement of next-generation sequencing (NGS) and mass spectrometry instruments, proteogenomic approaches have facilitated the identification of new protein-coding regions, translatome initiation sites, sequence variations, selective splicing, and various other genomic variations [6]. Currently, the most widely adopted method involves exome sequencing of tumor cells to uncover mutations, leading to the discovery of neoantigens. However, recent studies have revealed that a substantial portion of neoantigens arises from post-translatome erroneous splicing events and degradation transport processes [7]. Many translatome-derived proteins or peptides exhibit high affinity for the Major Histocompatibility Complex. Consequently, the translatome holds significant potential for mining variant information and guiding individualized neoantigen identification. Transcriptome sequencing, which involves sequencing all mRNA in tissues or cells, whether or not they are undergoing translatome, cannot ascertain whether previously considered non-coding RNAs can be translated. RNC-mRNA sequencing directly investigates biological phenotypes at the translatome level and reveals which genes are actively translated and the efficiency of translatome initiation which profoundly contributes to deepening transcriptome research. It can rectify the problem of too few differentially expressed genes observed in transcriptome sequencing. In 2011, Schwanhausser et al. [8] published an article in Nature and revealed that translatome regulation accounts for a staggering 54% of the overall impact on the organism, surpassing the combined influence of other stages (mRNA generation, mRNA degradation, and protein degradation) through theoretical calculations and experimental methods. It rightfully holds the throne as the master regulator and a paramount facet of life regulation. Thus, studying neoantigens at the translatome level is absolutely essential.

In this study, our objective was to investigate and validate the anti-tumor effects of translatome-derived neoantigen peptide vaccines. We screened for non-synonymous cell mutations from the LLC cell translatome data and synthesized peptide vaccines based on these translatome-derived neoantigens. Finally, our findings demonstrate that lung cancer translatome-derived neoantigens can be recognized by the immune system, and that peptide vaccines based on these translatome-derived neoantigens exhibit significant anti-tumor effects in a murine model of lung cancer.

## Materials and methods

### Cell lines and animals

The mouse lung adenocarcinoma cell line LLC (BLUEFBIO, Shanghai, China) was cultured in RPMI 1640 (Gibco, 31,870,082, Beijing, China) supplemented with 10% fetal bovine serum (FBS), 1% penicillin–streptomycin. Cells were grown at 37 °C in humidified incubators containing an atmosphere of 5% CO_2_. This line is widely used as a model for metastasis and the mechanisms of lung cancer chemotherapeutic agents [9]. In compliance with public policies on animal research, female 5 weeks old C57BL/6 mice, with a body weight of 18±2g were obtained from SiPeiFu (Beijing) Biotechnology Co., Ltd. All animal experiments were carried out under specific pathogen-free conditions.

### RNC-mRNA extraction

With the aid of the TRIzol RNA extraction agent (Invitrogen, 12,183,555, Shanghai, China), ribosome-nascent chain (RNC)-mRNA was isolated following with the instruction of test kits.

### RNC-mRNA seq

As described previously [10], RNA was purified with RNA beads (Illumina, 20,040,532, SanDiego, USA) first. Libraries were constructed with Illumina TruSeq.

RNA Prep Kit v2 and sequenced on an Illumina HiSeq2000 for 50 cycles at.

Mingma (Shanghai) Biotechnology Co., LTD. (Shanghai, China). For the sequence analysis, only high-quality reads that passed the Illumina quality filters were preserved.

### Translatome-derived neoantigen analysis and selection

Translatome sequencing involves the sequencing of RNC-mRNA within biological samples and conversion of the data into readable sequence information [11]. Initially, both transcriptome sequencing and translatome sequencing were performed on LLC cell lines. Subsequently, the translatome sequencing files were aligned to the mouse genome (GRCm38) using the STAR-2.6.0a tool [12]. The analysis and selection process proceeded as follows:

#### Mutation data analysis

Translatome mutation analysis refers to the process of analyzing mutations within the translatome sequencing data, aiding researchers in understanding variations in gene expression. Sentieon, an acceleration analysis software based on GATK, was utilized for translatome mutation analysis.

Mutated reading frames were obtained from non-coding regions. In essence, this process involves selecting sequences from the mutation analysis annotation results that include RefSeq numbers and the mutated cDNA positions. Subsequently, the NCBI's ORFFinder tool is employed to predict the reading frame, thereby determining the position of the mutation within the reading frame. Finally, the mutated reading frame is synthesized.

Protein sequences corresponding to wild-type and mutant reading frames were obtained. Based on the position of the mutations within the reading frames, we calculated their positions within the protein sequences and extracted wild-type and mutant peptide chains of 25–31 amino acids in length (synonymous mutations, where the wild-type and mutant reading frames were the same, were excluded; reading frames with protein sequences shorter than 25 amino acids were also filtered out).

#### Expression analysis

Expression analysis entails the examination of RNA sequencing data to study the expression levels of different genes across various samples and their variations [13].

#### MHC typing analysis

MHC typing analysis based on sequencing data involves the analysis of the MHC genomic region using sequencing techniques to determine the genotypes and allele information of MHC molecules.

#### Affinity analysis and final scoring

The NetMHCpan software was employed to predict the binding affinity between peptides and MHC molecules [14]. Ultimately, a comprehensive priority score was calculated based on affinity mutant score, expression score, and allele frequency (priority score = affinity mutant score × expression score × allele frequency). The top 10 sequences with the highest priority scores were selected as our target neoantigen sequences.

### Peptide synthesis and adjuvant selection

We employed standard solid-phase peptide synthesis with stringent quality control (purity > 90%) to synthesize ten peptides. Polyinosinic-polycytidylic acid (Poly(I:C)) was selected as the adjuvant.

### Mouse immunization

C57BL/6 mice were depilated in the inguinal regions. Using an insulin syringe, 200 μL of cell suspension (5 × 10^5^ cells/each) was injected into the left inguinal region of each mouse. Tumor formation was monitored every other day. Visible subcutaneous tumors appeared approximately 6–7 days later. A total of 16 mice that successfully developed tumors of similar sizes were divided into four groups: PBS group, Poly I:C group, routine peptide group, and translatome peptide group with four mice in each group. The adjuvant, Poly(I:C) at a dose of 50 μg, was mixed with a 50μL (2 mg) dose of peptide solution and supplemented with PBS to a final volume of 200μL per mouse to prepare the therapeutic vaccine.

Treatment began when tumor sizes reached 40–70mm^3^ (approximately 1 week after tumor implantation), designated as day 0. Mice were injected with the vaccine (200 μL/mouse) in the right inguinal region on days 0, 9, and 16, totaling three injections.

Starting from day 0, tumor growth was measured every three days (days 3, 6, 9, 12, 15, 18) using calipers, and tumor volume was calculated using the formula: tumor volume (mm^3^) = *D* × *d*^2^/2, where D and d represent the longest and shortest diameters, respectively.

The bone marrow-derived dendritic cells (BMDCs) were generated according to a method described previously [15]. BMDCs were cultured in RPMI1640 medium supplemented with 1% penicillin/streptomycin, 10% FBS, and 20 ng/mL GM-CSF (BD Pharmingen, 564,747). Non-adherent cells were collected as immature BMDCs for subsequent experiments on day 6.

### IFN-*γ* ELISpot

Enzyme-linked immunospot (ELISpot) analysis was conducted using a mouse IFN-*γ* ELISpot kit (BD, 552,569, San Jose, CA, USA) according to the manufacturer's instructions. In brief, dendritic cells (DCs) (5 × 10^4^) were stimulated with the neoantigen peptides (4 µg each) under 5% CO2 at 37 °C for 24 h. Subsequently, 3 × 10^6^ splenocytes were co-cultured with DCs (5 × 10^4^) at 37 °C for 36 h to detect cytokine secretion. All samples were tested in triplicates.

### The inflammatory cytokines release assay

The levels of inflammatory cytokines in the serum, such as IL-12p70, IL-6, IL-10, tumor necrosis factor (TNF), IFN-*γ*, and monocyte chemotactic protein 1 (MCP-1), were determined through ELISA, according to the manufacturer’s instructions (Bio Legend, San Diego, CA). Measurements were performed in triplicates.

### In vivo antitumor effect of neoantigen vaccine

To study the mechanism of the antitumor effects of the neoantigens, mouse splenocytes after euthanizing the mice on day 18 were extracted for flow cytometry analysis to determine the proportion of CD3 (Invitrogen, 11-0031-82, Shanghai, China) and CD137(Invitrogen, 25-1371-82, Shanghai, China). Cells were incubated for 25 min at 4 °C, stained for the antibodies mentioned above, and diluted in PBS containing 1% BSA and 5 mmol/L EDTA at a ratio of 1:100, according to the manufacturer’ s protocol. Cells were analyzed by BD FACS Canto flow cytometry. The data were analyzed using FlowJo V10 (Ashland, OR, USA).

Simultaneously, tumor tissues from the mice euthanized on Day 18 were fixed with polyformaldehyde solution for immunofluorescence staining and analysis. By observing the distribution of CD3+/CD137+ cells, we assessed the infiltration of effector T cells within the tumor tissue.

### Statistics

The data of flow cytometry were analyzed using FlowJo V10. The tumor sizes were determined using a caliper and the formula (*A* × *B*^2^)/2. All analyses were two tailed test and carried out using GraphPad Prism 5.03 (San Diego, CA, USA/http://www.graphpad.com). Values of ∗ *p* ≤ 0.05 were considered statistically significant.

## Results

### Analysis of translatome-derived neoantigens

**Fig. 1:**
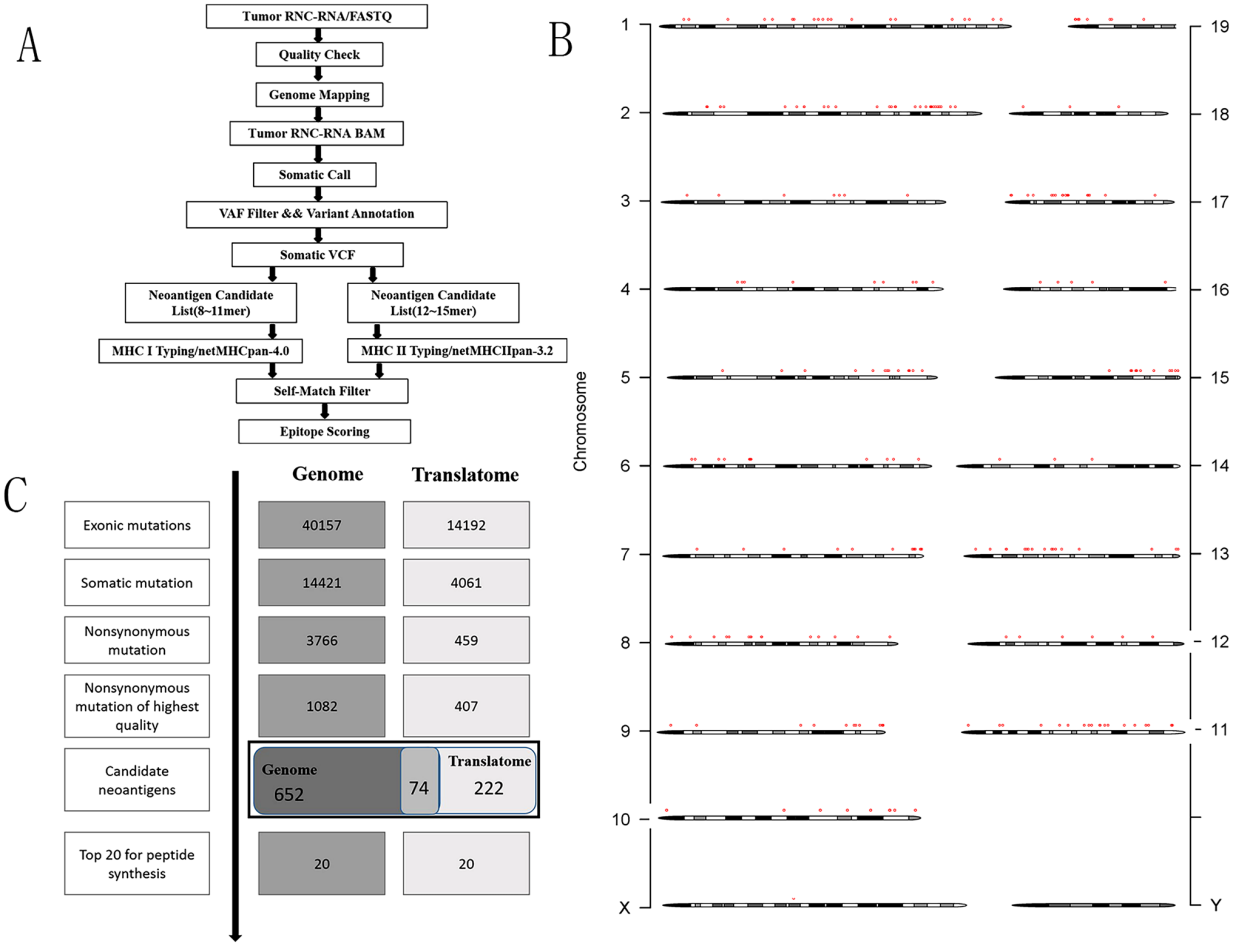
(**A**) Schematic of the workflow described in the analysis of translatome-derived neoantigens. (**B**) The chromosomal positions of the neoantigens. (**C**) The number of mutations obtained during neoantigen analysis

Translatome sequencing was performed on LLC cells to predict MHC-I-restricted neoantigens based on somatic mutations and MHC typing information. Subsequently, the obtained 31-mer peptide sequences were subjected to affinity analysis using software such as NetMHCpan and NetMHC-IIPAN. These analyses were complemented with tumor transcriptome information to assess the expression of the neoantigens. Ultimately, a comprehensive scoring system was employed, considering affinity, presentation, mutation frequency, to select the top 10 candidate neoantigen peptides, as shown in Fig. 1A. During the candidate antigen analysis process, the mutation filtering results are shown in Fig. 1B, and the finally obtained neoantigen chromosome distribution is shown in Fig. 1C. In Table 1, the candidate antigen peptides are listed by number, along with their respective neoantigen names, MHC types, amino acid changes, gene names, mutation positions, priority scores, and mutation sequences. The neoantigens based on routine analysis [16] as controls are listed in Table 2.

**Table 1:** Translatome-derived Neoantigens in Lung Cancer

Neo_Antigen_ID	MHC_allele	Amino_Acid_Change	Gene_Symbol	peptide_position	priority_Score	Augmented_peptide
FRNC57LLC20220811-Mettl25-1	H-2-Kb (M1)	A/P	Mettl25	5	92	DRLCYLKEQDGVAWSPLVKLFDPVQSPRCYA
FRNC57LLC20220811-Rasa4-2	H-2-Kb (M3)	G/V	Rasa4	9	89	NNRGLLRSYHPGIFRVDKWSCCHQKDKTDQG
FRNC57LLC20220811-Trappc11-3	H-2-Kb (M5)	S/N	Trappc11	6	83	HQLLFVRHQFKIAFFNELKQDTQNALKNYRI
FRNC57LLC20220811-Wdr3-4	H-2-Db (M7)	V/M	Wdr3	2	83	GSIMREGRDRVVNLAMDKTGRILACHGTDSV
FRNC57LLC20220811-Cog3-5	H-2-Kb (M9)	I/V	Cog3	6	81	REQIAPFHTEFTIKEVSLDLKKTRDAAFKIL
FRNC57LLC20220811-Zfp775-6	H-2-Db (M11)	T/A	Zfp775	4	80	SCPSRAALRAHQRVHAAAELLRSQSAVRDGV
FRNC57LLC20220811-Hjurp-7	H-2-Kb (M13)	S/R	Hjurp	2	78	MGRQDRRLHQQLKESRSRFQTLMKRLIAKYN
FRNC57LLC20220811-Nr2c1-8	H-2-Db (M15)	T/I	Nr2c1	9	78	RNGDTSFGAFHHDIQINGDVSRAFDTLAKAL
FRNC57LLC20220811-Zfp777-9	H-2-Kb (M17)	S/L	Zfp777	8	77	GYASPERGSAFNPKHLLKPRPKSPSSGSGGG
FRNC57LLC20220811-Lrrc28-10	H-2-Db (M19)	Q/H	Lrrc28	6	77	IGGLRALRHLRLANNHLQFLPPDFGSETLST

**Table 2:** Routine-derived Neoantigens in Lung Cancer

Neo_Antigen_ID	HLA_allele	HLA_allele	Gene_Symbol	peptide_position	priority_Score	Augmented_peptide
1	H-2-Db	H-2-Db	Smtn	9	100	VEAPVSSEPLPHPLEAPSPEPPMSPVP
2	H-2-Kb	H-2-Kb	Eya3	1	100	AHILSVPVSETTYSGQTQYQTLQQSQP
3	H-2-Db	H-2-Db	Emc1	9	100	LARDEFNLQKMMVMVTASGKLFGIESS
4	H-2-Kb	H-2-Kb	Leprot	7	99	VSAFGLPVVLARVGVIKWGACGLVLAG
5	H-2-Db	H-2-Db	Zmym1	2	99	ACSSSYNSAVMESSSVNVSMVHSSSKE
6	H-2-IAb	H-2-IAb	C77080	14	89	VLAAPAVAPGQVSAIDTSPASPSMPQT
7	H-2-IAb	H-2-IAb	Ewsr1	2	89	QAYSQPVQGYGTGAYDSTTATVTTTQA
8	H-2-IAb	H-2-IAb	Arid1a	6	88	QRTLLDPGRFTKVSSPAHTEEEEEEHL

### Synthesis of translatome-derived neoantigen peptides and immunogenicity assessment

**Fig. 2:**
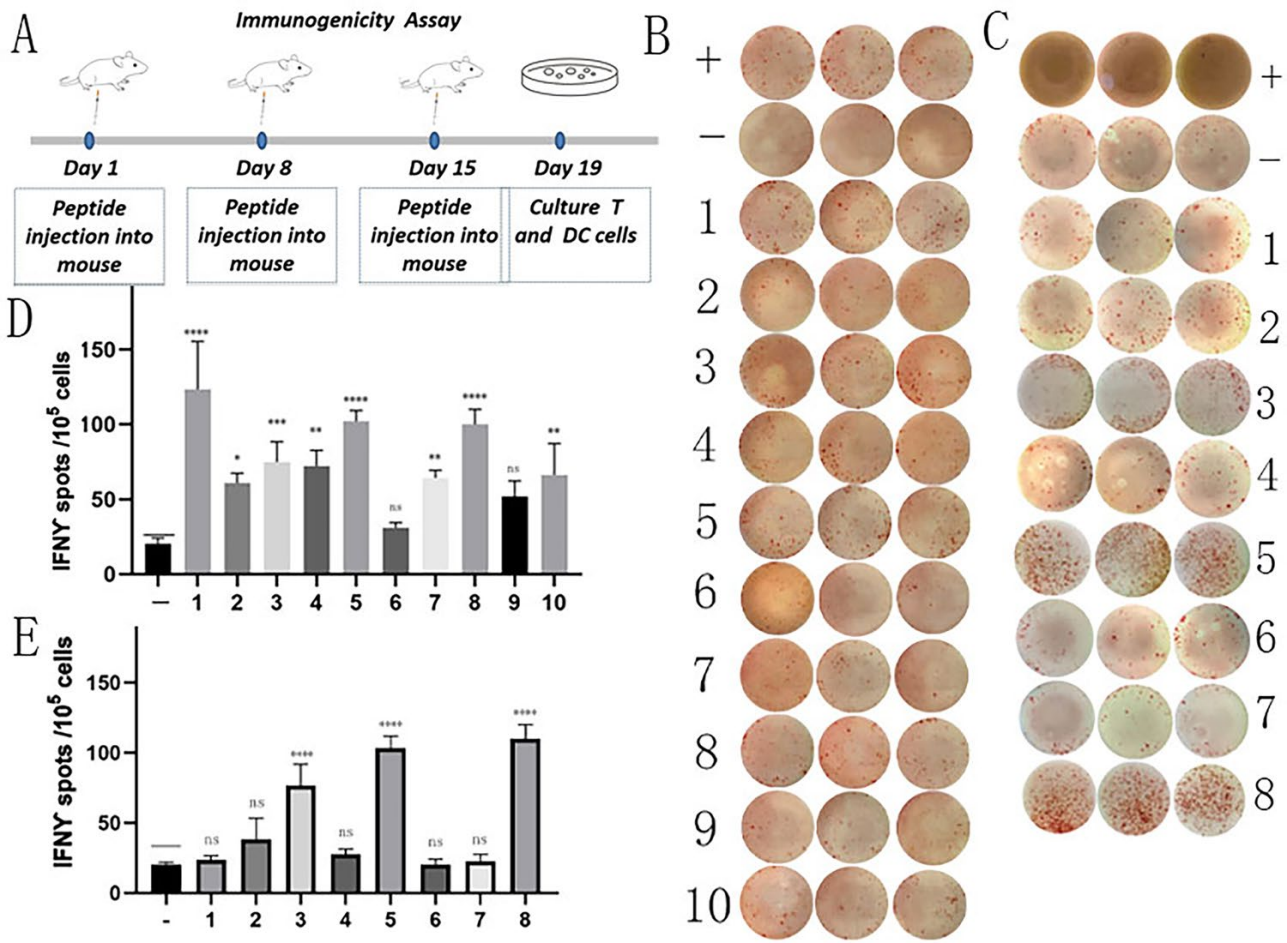
Immunogenicity assessment of neoantigen peptides. (**A**) C57BL/6 mice were subcutaneously immunized with individual neoantigen peptides on days 1, 8 and 15. Immunogenicity of the neoantigen peptides was assessed on day 19 using ELISpot. (**B**) The ELISpot assay spot images of translatome-derived neoantigen peptides. (**C**) The ELISpot assay spot images of routine neoantigen peptides. (**D**) Statistical results of the number of spots detected in the ELISpot assay of translatome-derived neoantigen peptides. (**E**) Statistical results of the number of spots detected in the ELISpot assay of routine neoantigen peptides. The data are presented as the means ± s.e.m. from three independent experiments. ****P < 0.0001, ***P < 0.001, **P < 0.01, *P < 0.05 and ns were obtained for the comparison of IFN-γ production by PBMCs stimulated without a peptide. ns, no statistical difference.

To assess the immunogenicity of the aforementioned 10 neoantigen peptides, we initially performed three subcutaneous immunizations on ten C57BL/6 mice using each of the 10 peptides separately. Subsequently, splenocytes were extracted and subjected to in vitro ELISpot experiments to evaluate the secretion levels of IFN-*γ* (Fig. 2). The results indicated that eight of the new antigens, namely 1 (Mettl25), 2(Rasa4), 3 (Trappc11), 4 (Wdr3), 5 (Cog3), 7 (Hjurp), 8 (Nr2c1), and 10 (Lrrc28), induced significant immune responses. The routine neoantigen peptides, namely 3, 5, 8, induced significant immune responses. These above positive peptides were used for anti-tumor experiments in vivo.

### In vivo antitumor study of translatome-derived neoantigen peptide vaccine

**Fig. 3:**
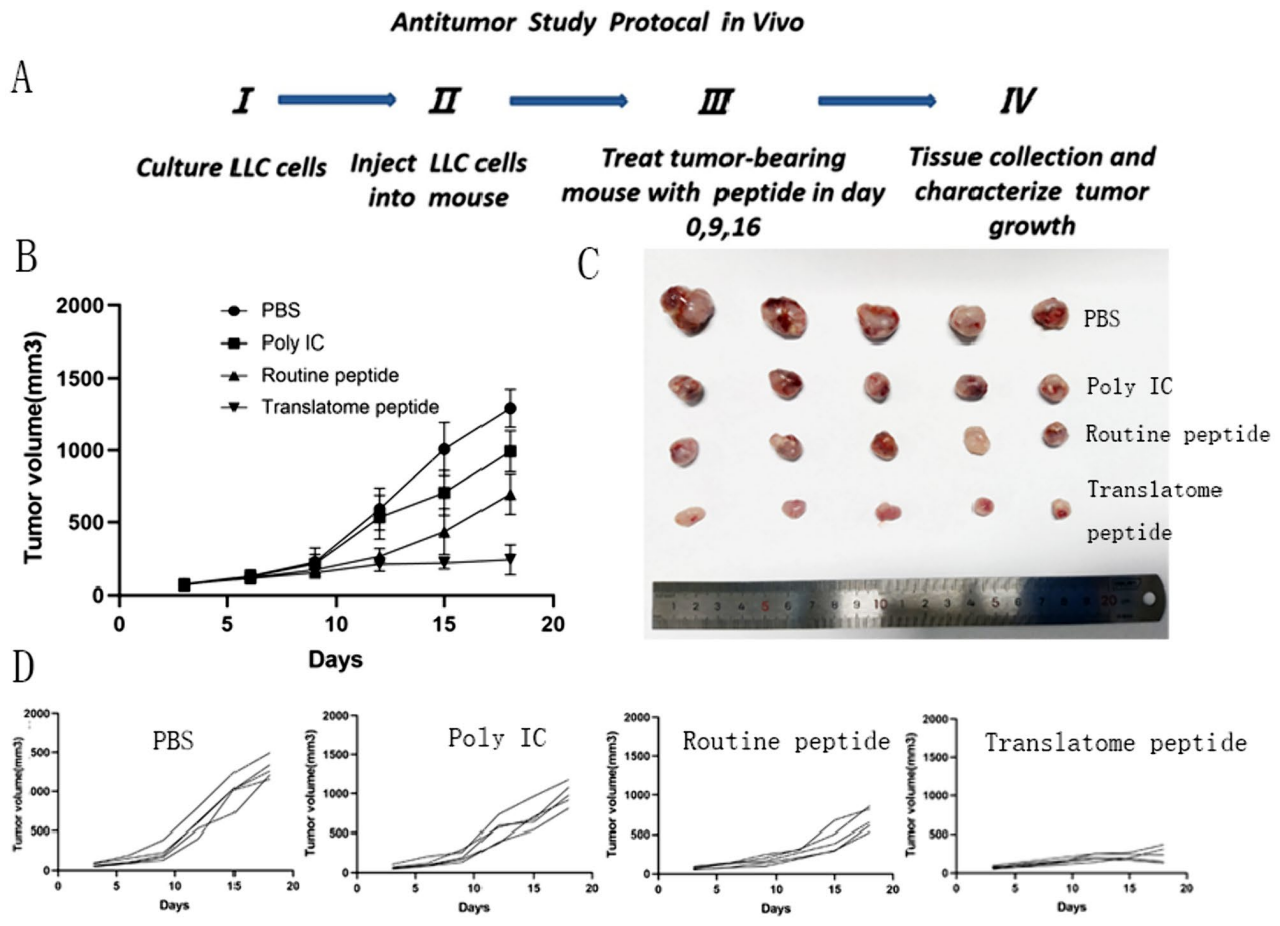
Subcutaneous Tumor Model Establishment, Treatment, and Tumor Volume Tracking. (**A**) Lewis cells were transplanted into C57BL/6 mice. Treatment began on Day 0, with injections administered on days 0, 9, and 16 to the four groups of mice. Starting from day 0, tumor growth was measured every three days (days 3, 6, 9, 12, 15, 18) using calipers. On day 18, the mice were euthanized. When tumors became palpable, the mice were randomized into four groups: PBS group (PBS), Poly(I:C) alone group (Poly IC), routine neoantigen peptide group (routine peptide), and the translatome-derived neoantigen peptide group (translatome peptide). (**B**), (**C**) and (**D**) The growth curves of average tumor volumes for all four groups

To further validate the antitumor effect of translatome-derived neoantigens, we prepared a neoantigen peptide vaccine using Poly(I:C) as an adjuvant. To investigate the synergistic anti-neoplastic effect, the therapeutic experiment in a mouse model was designed (Fig. 3A). We designed four treatment groups: PBS group (PBS), Poly(I:C) alone group (Poly IC), routine neoantigen peptide group (routine peptide), and the translatome-derived neoantigen peptide group (translatome peptide). We monitored and recorded changes in tumor volume in each treatment group with four mice and dissected the subcutaneous tumors for comparative analysis.

In in vivo experiments evaluating the treatment efficacy, mice injected with the translatome-derived neoantigen vaccine exhibited significant inhibition of tumor growth with a notable reduction in average tumor volume compared to the other group, because Poly(I:C) could promote recognition, uptake, and presentation of neoantigens, which enhances the immunogenicity of antigens and overcomes immune suppression against tumor antigens [17]. The anti-tumor effect of the routine peptide group and adjuvant-alone group was better than the PBS group. The growth curves of average tumor volumes for all four groups are illustrated in Fig. 3C and D.

From the dissected tumor blocks within the mice post-euthanasia (Fig. 3B), it is evident that the translatome-derived neoantigen group had smaller tumor volumes, more regular shapes, and smoother edges. In contrast, the PBS group exhibited noticeably rapid tumor proliferation, spreading to the surrounding areas and forming multiple clusters. The translatome-derived neoantigen group significantly inhibited the development of LLC subcutaneous tumors. Therefore, these results suggest that the translatome-derived neoantigen vaccine demonstrates a potent antitumor effect.

### Mechanistic study of anti-neoplastic effects of translatome-derived neoantigen peptide vaccine

**Fig. 4:**
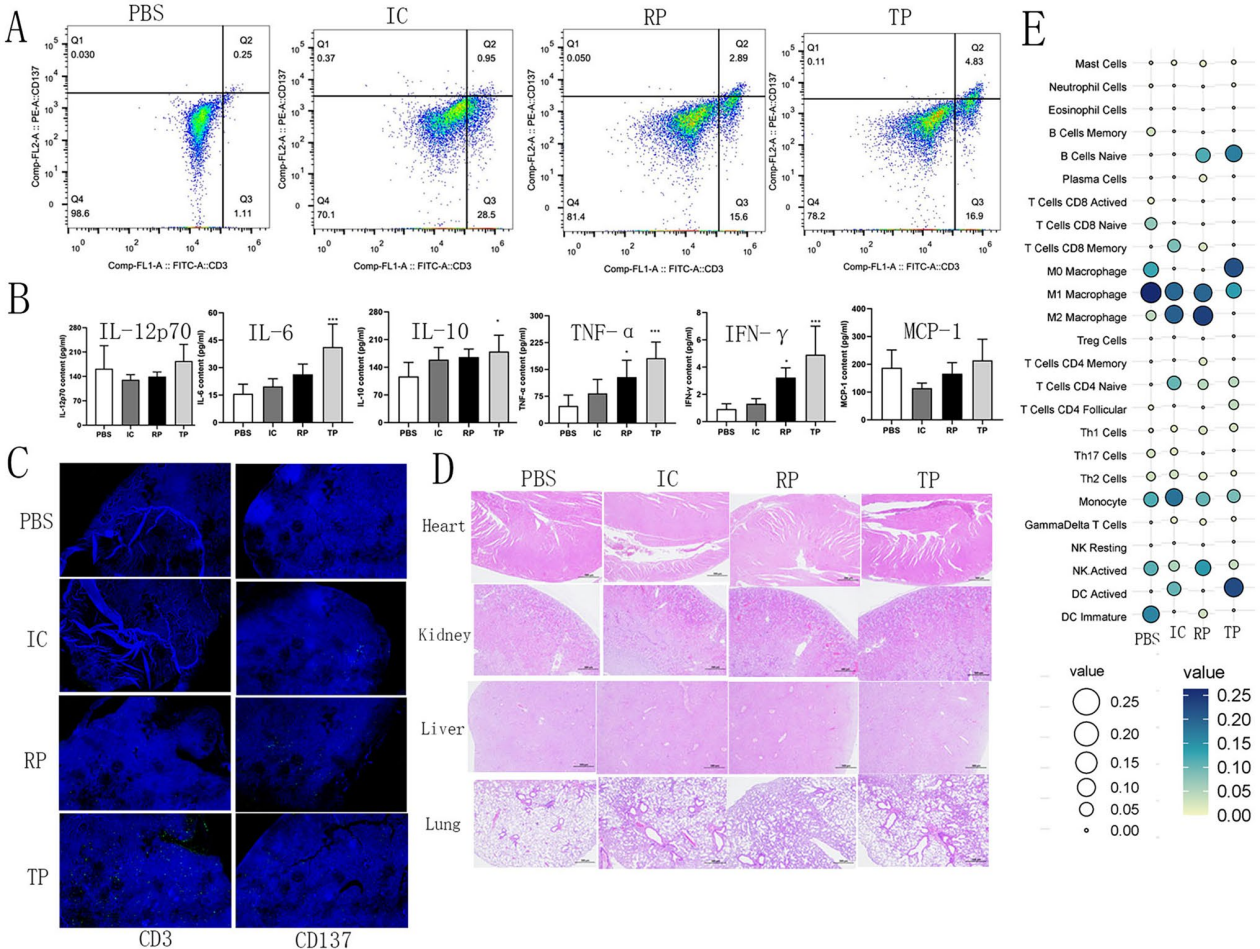
Antitumor immunity of neoantigen peptide in vivo. (**A**) The percentage of the CD3 + /CD137 + cells in the splenocytes. (**B**) ELISA assay of inflammatory cytokines in the serum samples from mice after treatment (*n* = 5). (**C**) Immunofluorescence analysis of tumor tissues from mice after treatment. On day 18, tumor tissues were resected from the mice. In the IF analysis, a combination of anti-CD3 antibody and anti-CD137 antibody (green) was used for primary staining. The nuclei were stained with DAPI (blue). Microscopic examination of IF samples was conducted at 40 × magnification. (**D**) H&E staining of heart, liver, kidney and lung of the mice after treatment. Microscopic examination of H&E at 40 × magnification. (**E**) Immune cell analysis of tumor tissues by mRNA sequencing. Results represent 1 of 2 independent experiments. IC, Poly IC; RP, routine peptide; TP, translatome peptide

T cells play a pivotal role in anti-tumor immunity by recognizing and attacking tumor cells and participating in the immune monitoring and clearance of tumors [18]. The assessment of activated T cells proportions within splenic cells revealed percentages of CD3+/CD137+ T cells in the PBS, Poly IC, routine peptide, and the translatome peptide groups to be 0.25, 0.95, 2.89 and 4.83%, respectively (Fig. 4A), concurrently confirming the marked capability of the translatome-derived neoantigen peptide vaccine to inhibit tumor growth.

Research suggested that escalated levels of inflammatory cytokines in the serum, such as IL-12p70, IL-6, IL-10, tumor necrosis factor (TNF), IFN-*γ*, and monocyte chemotactic protein 1 (MCP-1), lead to improved responsiveness to anti-PD-1 inhibition and prolonged survival in NSCLC [19, 20]. Our experimental results demonstrated a collective augmentation in levels of all six cytokines subsequent to the combination of the translatome-derived neoantigen peptide and Poly(I:C) therapy (Fig. 4B).

Immunofluorescence analysis of tumor tissue (Fig. 4C) showed a greater presence of positive cells in the tumor tissue of mice treated with the combination of the neoantigen peptide and Poly(I:C), indicating a higher and deeper T cell infiltration. In contrast, the other treatment groups exhibit relatively fewer T cell infiltrations, which are concentrated in the shallower peripheral regions of the tumor tissue. This suggests that the combination of the neoantigen peptide and Poly(I:C) significantly enhances T cell infiltration.

Furthermore, to substantiate the safety of the neoantigen peptide, we performed pathological analyses on the heart, liver, lungs, and kidneys. The results revealed no immunologically adverse events in these organs across all four groups (Fig. 4D), indicating the relatively high safety profile of the combined therapy involving neoantigen peptide and Poly(I:C).

In the model, it was interesting to observe increased M1/M2 ratio in the neoantigen peptide group. In the macrophage subset, substantial disparities were evident, characterized by a marked elevation in levels of M1-like macrophages and a pronounced reduction in levels of M2-like macrophages in the neoantigen peptide treatment group, consequently leading to a significant increase in the M1:M2 ratio (Fig. 4E).

## Discussion

Over the past decade, an abundance of research in tumor immunotherapy has significantly enhanced our comprehension of the immune system's role in cancer. Recently, numerous novel immunotherapies have been increased cancer patients' survival rates. Immunotherapy based on tumor neoantigens effectively heightens patients' anti-tumor immune responses while diminishing off-target treatment toxicity [21]. However, further investigations are required to substantiate the effectiveness of this approach.

Advancements in Next-Generation Sequencing (NGS) technology and mass spectrometry instruments have empowered researchers to employ proteogenomics method for scrutinizing individual amino acid variations in cancer. Proteogenomic methods aid in discerning novel protein-coding regions, new translation initiation sites, sequence variations, selective splicing, and various other sources of genomic variation [22]. The proteomic and integrated analysis with genomic data will contribute to the identification of novel antigenic peptides. The ribosome, as a principal component of the central dogma, serves as a pivotal node in the flow of genetic information participating both in mRNA input and protein output. Across different species, the correlation between mRNA and protein abundance has been less pronounced. It has been posited that translating RNC-mRNA may provide a more accurate reflection of protein abundance for years [23, 24]. Nevertheless, there remains no consensus on this matter as evidenced by studies involving yeast, HEK293 cells, and tumor cells [25–27]. Nonetheless, these findings suggest that a broad and diverse array of translation regulations may occur at the three stages of translation—initiation, elongation, and termination—and within the spatial conformation of mRNA [28]. However, studies on translation kinetics reveal that the predominant factor influencing this regulation is typically translation initiation, as it determines the proportion of mRNA molecules that undergo translation [29]. As an upstream process in functional protein production, environmental and physiological changes provide a rapid and specific foundation for translation. Consequently, transient alterations in the global genomic translation state are frequently observed in non-steady-state research systems, such as cell differentiation, T-cell activation, and stress responses [30]. For instance, translation initiation in yeast is globally suppressed within minutes of being transferred to a non-fermentable carbon source, independent of mRNA levels [31]. This signifies that translation initiation is a pivotal factor with limited correlation between transcription and protein abundance. This is also associated with other known factors, such as protein degradation and elongation rate. When comparing the relative abundance of different cell populations under stable conditions, the effects of translational control are more readily observable. For example, in a study focused on the epithelial–mesenchymal transition (EMT) induced by transforming growth factor, changes in ribosome-bound mRNA were found to correlate with phenotype-related differential translation genes [32]. We hypothesized that there exists a strong correlation between RNC-mRNA and protein abundance in cells under stable conditions when considering multiple variables based on these findings. In this study, we used high-throughput sequencing and bioinformatics analysis. Leveraging tumor mutation abundance and MHC-peptide affinity as our guiding criteria, we tailored individualized T-cell epitopes, thereby reducing the pool of candidate antigenic peptides. Subsequently, we identified and prepared a lung cancer neoantigen vaccine. Through interferon-gamma enzyme-linked immunospot assays in vitro, we demonstrated the robust immunogenicity of the translatome-derived neoantigens. Furthermore, we validated the potent anti-tumor effects of the translatome-derived neoantigen vaccine based on a C57BL/6 mouse model of lung cancer subcutaneous tumors. Our findings were reinforced by immunofluorescence results, confirming an enhanced infiltration of T-cells within tumor tissues.

A significant challenge in tumor immunotherapy lies in the presence of intratumoral heterogeneity. Hence, exploring neoantigens from diverse sources, comparing their immunogenicity, and assessing their anti-tumor effects are crucial. The selection of a broader and superior array of neoantigens and targeting multiple neoantigens may mitigate tumor immune escape and enhance anti-tumor responses contributed to reducing the chances of resistance development to neoantigen-based immunotherapy. Currently, the most widely employed method for neoantigen selection relies on exome sequencing of tumor cells to identify mutations. Neoantigen immunotherapies have demonstrated objective efficacy only in a small subset of patients with documented responses. Therefore, substantial improvements are necessary to enhance clinical outcomes, including refining the accuracy and diversity of neoantigen prediction, overcoming immune evasion, and addressing tumor heterogeneity issues. These obstacles must be surmounted to achieve effective immune responses based on tumor-specific neoantigens and to offer a safe and efficacious solution for lung cancer patients. Due to tumor heterogeneity and the significant variability in the presentation of neoantigens across various tumor types, the discovery of targetable cancer neoantigens remains limited. Only approximately 10% of non-synonymous tumor cell mutations generate mutant peptides with high MHC affinity and only 1% of MHC-binding peptides are recognized by patient T cells according to DNA sequencing [33, 34]. Recent studies have uncovered a substantial number of neoantigens originating from translatome sources of proteins or peptides. Therefore, the incorporation of translatome through sequencing to identify tumor neoantigens will enrich the avenues for neoantigen discovery, potentially ushering in new possibilities to enhance the efficacy of neoantigen-based cancer vaccines in the future.

The limitations of our study include the following. First, we employed only one type of cell for the profiling of translatome-derived neoantigens. To establish more robust evidence, further validation with a variety of tumor cells is essential. Second, our study focused exclusively on translatome mutations as the source of neoantigens. Investigating neoantigens originating from translatome variations such as alternative splicing and modifications is also highly worthy of further research.

## Conclusion

Our research underscores that translational profiling sequencing has significant potential for mining mutation information and guides individualized neoantigen identification. When combined with advanced bioinformatics analysis techniques, this approach can select personalized neoantigens efficiently and feasibly for practical applications in a clinical setting. Neoantigen-based personalized tumor immunotherapy shows a promising outlook, promotes advancements in translational immunotherapy research based on neoantigens.

## Data Availability

All data are available upon request to correspondence author.
